# Neuroprotective Effects of Qingnao Dripping Pills Against Cerebral Ischemia *via* Inhibiting NLRP3 Inflammasome Signaling Pathway: *In Vivo* and *In Vitro*

**DOI:** 10.3389/fphar.2020.00065

**Published:** 2020-02-20

**Authors:** Chen Fu, Xinyang Zhang, Zixiu Zeng, Yang Tian, Xianglan Jin, Fengli Wang, Zhenmin Xu, Baoxin Chen, Hong Zheng, Xuemei Liu

**Affiliations:** ^1^ Central Laboratory, Dongfang Hospital, Beijing University of Chinese Medicine, Beijing, China; ^2^ Department of Traditional Chinese Internal Medicine, Beijing University of Chinese Medicine, Beijing, China; ^3^ Neurology Department, Dongfang Hospital, Beijing University of Chinese Medicine, Beijing, China

**Keywords:** cerebral ischemia, NOD-like receptor family pyrin domain containing 3 inflammasome, inflammatory response, apoptosis, middle cerebral artery occlusion, Qingnao dripping pills

## Abstract

Ischemic stroke patients suffer from relatively limited treatment options. Studies have shown that in cerebral ischemia, NOD-like receptor family pyrin domain containing 3 (NLRP3) inflammasome is a key mediator in mediating inflammatory responses and results in activation of apoptosis signaling pathways. Here we assessed the *in vivo* and *in vitro* effects of Qingnao Dripping Pills (QNDP), a traditional Chinese prescription, on inflammatory responses and apoptosis. Our results showed that QNDP could significantly decrease cerebral ischemia injury, improve neurological function and inhibit apoptosis in rats impaired by middle cerebral artery occlusion (MCAO). Further, we found that QNDP inhibited NLRP3 inflammasome expression both in MCAO rats and in SH-SY5Y cells under OGD. Moreover, the levels of inflammatory cytokines including interleukin-1β (IL-1β) and IL-18, which mediated by NLRP3 inflammasome and increased in MCAO rats, could be reduced by QNDP, suggesting that QNDP could protect the neurons against inflammation through a mechanism mediated by NLRP3 inflammasome. Nuclear factor-kappa B (NF-κB) was also involved in the anti-inflammatory effect of QNDP. In conclusion, QNDP had neuroprotective effects against cerebral ischemia *via* inhibiting NLRP3 inflammasome signaling pathway, and was a potential candidate for the future treatment of ischemic stroke.

## Introduction

Stroke is a leading cause of mortality and permanent disability globally, and the economic costs and post-stroke care are substantial (Collaborators, 2019). However, there are still relatively limited treatment options for ischemic stroke patients, largely because the molecular and cellular mechanisms underlying continue to be poorly understood (Wang et al., 2019). Recently, previous studies have found increasing amounts of evidence showing the critical role played by the inflammasome in cerebral ischemia (Gao et al., 2017).

The inflammatory response and apoptosis are essential processes which can cause cellular damage and cell death after stroke (Radak et al., 2017; Pan et al., 2018; Jayaraj et al., 2019). It has previously been observed that in response to cerebral ischemia, NOD-like receptor family pyrin domain containing 3 (NLRP3) inflammasome may be a key mediator in mediating inflammatory responses and result in activation of apoptosis signaling pathways (Guo et al., 2016; Pan et al., 2018; Hong et al., 2019). NLRP3 inflammasome is a large multi-protein complex that is composed of NLRP3, the adaptor apoptosis-associated speck-like protein containing a CARD domain (ASC), and pro-caspase-1(Banoth and Sutterwala, 2017). After assembly and activation of NLRP3 inflammasome, pro-caspase-1 clustering will permit autocleavage and formation of the active caspase-1(Lamkanfi et al., 2007). Moreover, the active caspase-1 converts interleukin-1β (IL-1β) and interleukin-18 (IL-18) into their mature forms to engage in immune defense (Guo et al., 2015; Gao et al., 2017), and also initiates activation of apoptosis, which is involved with Caspases family and B-cell lymphoma-2 (Bcl-2) Family and results in DNA fragmentation, degradation of cytoskeletal and nuclear proteins, cross-linking of proteins death receptor ligation, and lysosomal protease activation(Radak et al., 2017; Pan et al., 2018). Thus, it is necessary and meaningful to identify a drug that can control NLRP3 inflammasome activation in cerebral ischemia.

Qingnao dripping pills (QNDP) is a traditional Chinese prescription consisting of *Gardenia jasminoides* Ellis (Chaozhizi), *Panax notoginseng* (Burk.) F. H. Chen (Sanqi), and borneol (Bingpian). Its main functions include resuscitation, clearing heat, dredging meridians, and treating stroke caused by phlegm and blood stasis, dysphasia, hemiplegia, and facial paralysis (Zeng et al., 2019). A clinical study in China found that QNDP could ameliorate the neurological function and alleviate a series of symptoms in stroke patients, such as headache, thirst, constipation, etc. (Han et al., 2007). In addition, our previous work has shown QNDP could reduce infarct size in rat models of middle cerebral artery occlusion (MCAO) by mediating the levels of inflammatory factors (TNF-a, IL-1β, and IL-10) and inhibiting the mitogen-activated protein kinase (MAPK) signaling pathway, which is associated with inflammation and apoptosis(Liu et al., 2016; He et al., 2019a; Zeng et al., 2019). However, the effect of QNDP on NLRP3 inflammasome has not been explored at present.

In the present study, we assessed the potential roles of QNDP on NLRP3 inflammasome in cerebral ischemia. We demonstrate that QNDP could reduce inflammatory response and apoptosis *via* inhibiting NLRP3 inflammasome signaling pathway in cerebral ischemia *in vivo* and *in vitro*, further suggesting that QNDP is a novel treatment candidate for ischemic stroke.

## Materials and Methods

### Drug Preparations

QNDP is a specification of 0.038 g/pill, which is composed of *Gardenia jasminoides* Ellis (stir fried fruit), *Panax notoginseng* (Burk.) F. H. Chen (root), and *borneol* at a weight ratio of 70:30:1. *Gardenia jasminoides* Ellis (stir fried fruit) and *Panax notoginseng* (Burk.) F. H. Chen (root) were purchased from Hebei Ju Ren Tang Co., Ltd. (Hebei, China), and *borneol* was purchased from Guizhou Minzu Medicine Co., Ltd. (Guizhou, China). Raw drugs were authenticated by Professor Yuan Zhang according to Chinese Pharmacopeia 2015, morphologically and chemically. The voucher specimens were deposited in School of Chinese Materia Medica, Beijing University of Chinese Medicine and the scan of the vouchers were given in **Supplementary Table 1**. The voucher numbers for *Gardenia jasminoides* Ellis, *Panax notoginseng* (Burk.) F. H. Chen, and *borneol* specimens were 18-BUCM-Q3-1, 18-BUCM-Q1-2, and 18-BUCM-Q4-3, respectively.

The QNDP was produced by the Scientific Research Institute of Beijing Tong Ren Tang Co., Ltd. (Beijing, China). *Gardenia jasminoides* Ellis and *Panax notoginseng* (Burk.) F. H. Chen were extracted with ethanol, separated and purified, concentrated into dry powder, then added appropriate amounts of *borneol* to make the mixture. The QNDP was made by adding the polyethylene glycol 6000 and 4000 to the mixture. The clinical trial (No.2008L11182) of QNDP has been approved by the China Food and Drug Administration (CFDA) at 2008. *Geniposide* is the primary active constituent in QNDP, and it was the marker component for controlling the quality of QNDP by the company. According to the ultra-performance liquid chromatography (UPLC) analysis, the content of *Geniposide* (C_17_H_24_O_10_) should be no lower than 1.5mg/pill (**Supplementary Figure 1**).

The chromatography samples were prepared as follows: dissolve QNDP with 50% methanol according to 1:10, centrifuge to take 50 μL of the supernatant and added 450 μL of 50% methanol to dilute, then take 100 μL of the supernatant and added the mixture (methanol: acetonitrile = 1: 1) 300 μL, spin and mix for 60 s, centrifuge at 13000 rpm for 10 min, and take 100 μL for the liquid chromatography-tandem mass spectrometry (LC-MS) analysis.

### Animals

Adult male Sprague-Dawley rats were provided by Vital River Co. Ltd. (Beijing, China), weighing between 220 and 240 g. All rats were fed five per cage under controlled temperature (22 ± 2°C), with 12 h light/dark cycle and free access to food and water. The study was approved by the Animal Care and Use Committee of Dongfang hospital, Beijing university of Chinese Medicine.

### Materials and Reagents

Geniposide (lot number: 110749-201718), Ginsenoside Rg1 (lot number:110703-201832), Ginsenoside Re (lot number:1110754-201626), Notoginsenoside R1 (lot number: 110745-201619) were purchased from the National Institutes for Food and Drug Control (Beijing, China).

NLRP3 inflammasome inhibitor (MCC950) and 2% 2,3,5-triphenyltetrazolium chloride (TTC) solution were purchased from Sigma-Aldrich (USA). The primary antibodies were provided as follows: mouse monoclonal anti-Bad antibody (sc-8044), mouse monoclonal anti-ASC antibody (sc-271054) were purchased from Santa Cruz Biotechnology (USA). Rat monoclonal anti-BcL-XL (2764), anti-Caspase 1 (4199) were purchased from Cell Signaling Technology (USA). Rabbit polyclonal anti-IL-1β antibody (ab9722), rabbit polyclonal anti-IL-18 antibody (ab71495) and mouse monoclonal anti-NeuN antibody (ab104224) were purchased from Abcam (USA). Rabbit polyclonal Anti-NLRP3 antibody was purchased from Novus (USA). GAPDH (YM3029) was purchased from Immunoway (USA).

### LC-MS Analysis

One hundred microliter of the homogenate were used for detection using a QE-Orbitrap high-resolution mass spectrometer (Thermo, USA). Mobile phase A was 0.1% formic acid and 2 mmoL/L ammonium formatted in water, and mobile phase B was acetonitrile. The gradient elution procedure was as follows: 0–1.0 min, 30–60% B; 1.0–1.5 min, 60–90% B; 1.5–3.5 min, 90% B; 3.5–3.6 min, 90–30% B; 3.6–5.5min, 30% B. Other conditions of the procedure were as follows: analysis time, 0–5.5 min, 5 μL per injection; flow rate, 0.3 mL/min; the chromatography column was Thermo Hypersil Gold C18 (3 µm, 2.1×100 mm), the column temperature was 30°C, and the automatic sampler temperature was maintained at 4°C. They were detected using ESI in the negative ion modes. Other conditions were as follows: spray voltage, 3,000 V, evaporation temperature, 350°C; sheath gas, 40 Arb; auxiliary gas, 10 Arb; capillary temperature, 320°C; S-lens RF, 80, CE: 35.

### Cerebral Ischemia–Reperfusion Model

Cerebral ischemia–reperfusion was produced following the suture middle cerebral artery occlusion (MCAO) method and modified as previously described(Longa et al., 1989; Zeng et al., 2019). Briefly, rats were separated the branches of the left common carotid artery, and subjected to MCAO for 1.5 h and followed by 24 h. After surgery, rats were allowed to stay in their cages with free access to food and water. Before study, rats were randomly divided into sham group or MCAO group, and for MCAO group, they were further randomized to receive either QNDP or NLRP3 inhibitor or QNDP and NLRP3 inhibitor. In the sham group, this same operation was performed but no sutures were inserted. All rats were scarified at 24 h of reperfusion. In accordance with the previous experiments(Zeng et al., 2019), based on clinical usage, we used different doses of QNDP, the results showed that the effective dose of QNDP for rats was 0.15 g/kg (**Supplementary Figure 2**). QNDP was dissolved in (0.9%) saline solution for the experiments. Glycol 6,000 and 4,000 were dissolved by 0.9% sodium chloride as vehicle treatment.

Rats were randomly divided into five groups (eight animals each): (1) sham group (sham), administered glycol 6,000 and 4,000 (1 mL/100 g, i.g.). (2) MCAO group (MCAO), administered glycol 6,000 and 4,000 (1 mL/100 g, i.g.). (3) QNDP group (QNDP), administered QNDP (0.15 g/kg, i.g.) 2 h after surgery, and then once a day. (4) NLRP3 inhibitor group (NLRP3 inhibitor), administered NLRP3 inflammasome inhibitor MCC950 (100 nM, i.p.) 30 min prior to surgery, as a positive control, and then administered glycol 6 000 and 4 000 after surgery 2 h (1 mL/100 g, i.g.). (5) QNDP and NLRP3 inhibitor group (QNDP+ NLRP3 inhibitor), administered NLRP3 inflammasome inhibitor MCC950 (100 nM, i.p.) 30min prior to surgery and then administered QNDP (0.15 g/kg, i.g.) once a day.

### Evaluation of Neurological Deficits

The neurological deficits of MCAO rats after 24 h reperfusion were evaluated by an operator blinded to the experimental conditions. Neurologic deficits were evaluated with a modified method according to Garcia score (Garcia et al., 1995). The Garcia score, an 18-point neurobehavioral scoring system, was divided into 6 subjects, which includes spontaneous activity, movement symmetry of 4 limbs, symmetry of forepaw outstretching, climbing a wire cage, body proprioception and response to touching the vibrissae. Rats demonstrated normally were assigned as the highest score (18 scores) and the severe neurological impaired were assigned as the lowest score (3 score).

### Measurement of Infarct Volume

Twenty-four hours after reperfusion, rats were anesthetized by 10% chloral hydrate (3.5 mL/kg, i.p.), and the brains were immediately removed and frozen at −20°C for 15 min. Brains were cut into five 2-mm-thick coronal sections, incubated in 2% TTC at 37°C for 30 min in the dark, and then fixed in 4% paraformaldehyde solution at 4°C for 24 h. Brain slices were photographed and analyzed by ImageJ analysis software (version 6.0, NIH). The infarct volume was calculated as follows: the left infarct volume/whole brain volume × 100%.

### Brain Water Content

Brains were quickly weighed on the electronic analytical balance and recorded the wet weight. Then whole brains were dried at 110°C for 24 h to obtain the dry weight. The percent brain water content was calculated as follows: (wet weight-dry weight)/wet weight×100%.

### Cell Culture and Lentivirus Transfection

The human neuroblastoma cell line (SH-SY5Y cells) were purchased from the American Type Culture Collection. The cells were cultured in RPMI 1640 medium (Hyclone, Thermo Fisher Scientific) containing 10% fetal bovine serum (Hyclone) in a humidified atmosphere at 37°C with 5% CO_2_. Cells were incubated in neurobasal medium, supplemented with 2% B27 supplement (17504-044, Gibco) and 0.5 mM L-glutamine (35050-061, Gibco). 3 days of plating, cells were added 10 M retinoic acid (0695, Sigma) to the medium, and induced to differentiate into a homogeneous population of the cells with neuronal morphological structure (Kume et al., 2008). After 7–8 days, cells were used in the experiments.

The lentivirus for knock down NLRP3 and the control lentivirus (LV-scramble) was synthesized by Genechem (Shanghai, China). A plasmid expressing shRNA that targeting human NLRP3 was constructed using the GV248 vector. The shRNA target sequence was designed follows: 5′-GTGCGTTAGAAACACTTCA-3′. The shRNA (100 nmol/L) were transfected into the cells using Lipofectamine 2000 reagent (11668, Invitrogen) according to the manufacturer’s instructions. After 6 h of transfection, the medium was replaced by RPMI 1640 medium for 24 h and then treated with or without OGD deprivation.

### OGD Model

After transfection, the cells were exposed to oxygen–glucose deprivation. For OGD, cells were exposed to hypoxia (95%N_2_/5%CO_2_) at 37°C for 12 h and normal culture conditions (95% air, 5% CO_2_) for an additional 12 h. (**Supplementary Figure 3**). At the OGD same time, added different concentrations of QNDP into cells. The result showed that the effective concentration of QNDP was 5μg/mL for SH-SY5Y cells expose to OGD (**Supplementary Figure 4**). The SH-SY5Y cells were randomly divided into the following groups: (1) normal control group (CON); (2) OGD group OGD; (3) QNDP group (OGD+QNDP); (4) sh-NLRP3 in normal cells group (CON+ sh-NLRP3); (5) sh-NLRP3 under OGD deprivation group (OGD+ sh-NLRP3); (6) sh-NLRP3 under OGD deprivation (OGD+ sh-NLRP3) and treated with QNDP group (OGD+QNDP+sh-NLRP3).

### Cell Viability

CCK-8 assay was performed to evaluate cell viability of SH-SY5Y cells. The cells were seeded into 96-well plates at a density of 1×10^5^ cells per well for 24 h. In brief, CCK-8 buffer (10 μL) was added into each well. The cells were incubated 37°C for 4 h. The absorbance measurements were taken at 450 nm on a microplate reader (Bio-Tek, USA).

### Quantitative Real-Time Polymerase Chain Reaction (qRT-PCR)

Total RNA was extracted from left cerebral samples using TRIzol reagent (DP405-02, TIANGEN, China). cDNA was amplified by sequence-specific primers. Total RNA (1 μg) was reverse transcribed into cDNA using Reverse Transcriptase M-MLV Kit (2641B, TaKaRa, Japan). The qRT-PCR was performed with the ABI 7500 sequence detection system (Applied Bio-systems) using SYBR^®^ Premix Ex Taq™ II (Tli RNaseH Plus) (RR82LR, TaKaRa). Primer sequences for IL-1β and IL-18 were synthesized and obtained from Sangon Biotech (Shanghai, China). Primers were provided as follows: IL-1β: Forward, 5′-TCATTGTGGCTGTGGAGAAG-3′ and Reverse, 5′-CTATGTCCCGACCATTGCTG-3′; IL-18: Forward, 5′-GGATCTTGCGTCAATTCAAGG-3′ and Reverse, 5′-TTGGCTGTCTTTTGTCAACGA-3′. GAPDH: Forward, 5′-CCATCACTGCCACTCAGAAGA-3′ and Reverse, 5′-CATGAGGTCCACCACCCTGT-3′. The relative mRNA expression was quantified using the 2^−ΔΔCt^ method, GAPDH as housekeeping gene. All reactions were performed in triplicate.

### Immunofluorescent Staining

For images from *in vivo* brain tissues, brains were removed following MCAO, and post-fixed using 4% PFA. After preparing the frozen sections, the brain sections were cut into 15μm thick coronal slices. For images from *in vitro*, the SH-SY5Y cells were fixed by 95% acetone for 30 min, permeabilized by Triton X-100 for 10 min, blocked with 5% goat serum for 1h. Immunostaining was performed using rabbit anti-NLRP3 antibody (1:200 dilution) and mouse anti-NeuN antibody (1:1,000 dilution) overnight at 4°C. After washed by PBST, goat anti-mouse IgG H&L (Alexa Fluor^®^ 488) (1:1,000 dilution, Abcam) and goat anti-rabbit IgG H&L (Alexa Fluor^®^ 555) (1:1,000 dilution, Abcam) was incubated for 2 h at room temperature. Then, the cells or sections were incubated DAPI (10 μg/ml) for 5 min. Images were required by a fluorescence microscopy (BX71, Olympus, Tokyo, Japan).

### Western Blotting

The protein from the left cerebral tissues and SH-SY5Y cells was prepared and extracted as previously described (Zeng et al., 2019). Briefly, protein samples (40 μg/lane) were loaded in a 4–12% Bis-Tris SDS-PAGE, and then transferred to the 0.22 μm polyvinylidene fluoride (PVDF) membrane, and subsequently blocked with 5% non-fat milk for 1 h, incubated with the primary antibodies overnight at 4°C: Bad (1:1,000), Bcl-xL (1:1,000), cleaved caspase-3 (1:1,000), NLRP3 (1:1,000), ASC (1:500), cleaved caspase 1 (1:500), IL-1β (1:500), IL-18 (1:500). Membranes were washed with TBST three times and incubated with goat anti-rabbit IgG secondary antibody (1:5,000) for 1hour at the room temperature. GAPDH, or β-actin were used as internal control. Detection was performed with an Odyssey Infrared Imaging System (LI-COR, USA). Western blotting bands were analyzed using the ImageJ software (National Institutes of Health, USA). All experiments were performed in triplicate.

### Statistical Analysis

The date are shown as mean ± standard deviation (SD). Statistical analysis was used SPSS 25.0 statistical software. Statistical analysis was performed using Student’s t test and one-way ANOVA for comparisons between and among different groups. *P* < 0.05 was indicated statistically significant.

## Results

### Concentrations of the Major Effective Components of QNDP

According to LC-MS analysis, the identification of 4 major chemical ingredients of QNDP was performed, the mass spectrograms and chemical formulas of the main ingredients were shown in **Supplementary Figures 5** and **6**. According to the chromatograms results, their concentrations in QNDP were also determined as follows: Geniposide (145.6 ± 18.86) mg/g, Ginsenoside Rg1 (77.1 ± 2.4) mg/g, Notoginsenoside R1 (5.8 ± 0.2) mg/g, Ginsenoside Re (4.9 ± 0.3) mg/g (**Supplementary Table 2**).

### QNDP Was Neuroprotective *In Vivo*

**Figure 1:**
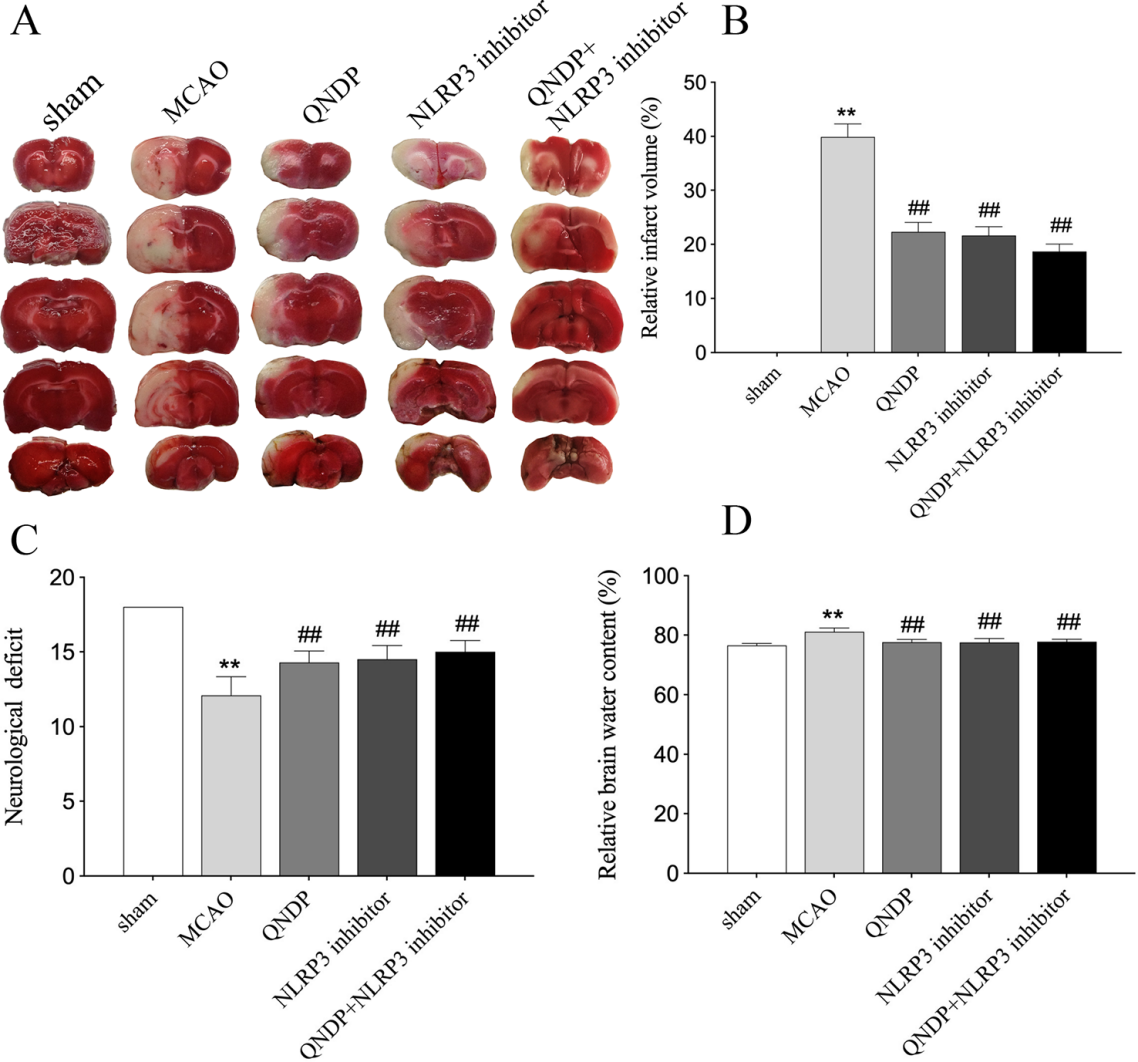
QNDP is neuroprotective in MCAO rats. **(A)** Images of cerebral infarction (white, infarct tissue; red, non-infarct tissue) were TTC-stained brain sections. **(B)** The infarct volume was quantified with Image J analysis and expressed as a percentage of the damaged ipsilateral hemisphere. **(C)** Neurological deficit by Garcia JH scores. **(D)** Brain water content. Data are presented as means ± SEM, n = 8 per group, **p < 0.01 compared with the sham group, ^##^ *p* < 0.01 compared with the MCAO group.

To investigate the neuroprotective effect of QNDP against cerebral ischemia injury, we examined the effect of QNDP on cerebral infarct volume, neurological scores and brain water content. As shown in **Figures 1A, B**, the quantitative assessment of TTC staining sections showed that MCAO group had an infarct volume of (39.89% ± 2.43%) (*p* < 0.01 vs. sham group). QNDP resulted in a significant decrease in cerebral infarct volume (22.32% ± 1.75%) as compared with MCAO group (*p* < 0.01). NLRP3 inhibitor group (21.65% ± 1.62%) and QNDP+NLRP3 inhibitor group (18.65% ± 1.37%) were also decreased in cerebral infarct volume, compared with MCAO group (*p* < 0.01). Neurological defects are shown in **Figure 1C**. The neurological scores in the QNDP group were significantly improved (*p* < 0.01) compared to the MCAO group. NLRP3 inhibitor group and QNDP+NLRP3 inhibitor group were increased in neurological scores, compared with MCAO group (*p* < 0.01). The brain water content in the MCAO group was significantly increased (*p* < 0.01) compared with the sham group. QNDP, NLRP3 inhibitor, QNDP+NLRP3 inhibitor markedly reduced the brain water content, data as shown in **Figure 1D** (*p* < 0.01). These results suggested that QNDP could significantly decrease cerebral ischemia injury and improve neurological function.

### QNDP Inhibited Apoptosis in Ischemic Cortex After Cerebral Ischemia *In Vivo*

**Figure 2:**
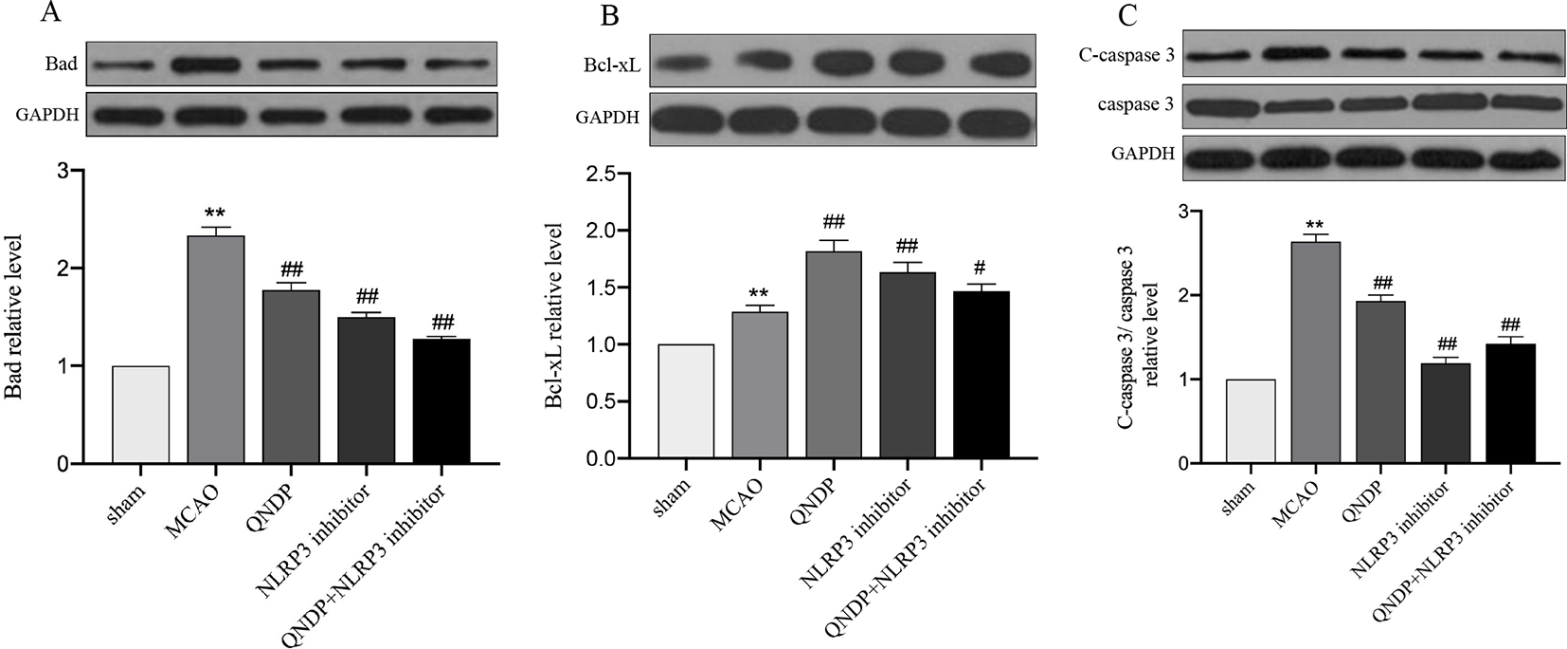
Effect of QNDP on the protein expression of Bad, Bcl-xL, and cleaved caspase 3/caspase 3 in MCAO rats. Representative immunoblots and quantification illustrating the effect of QNDP on protein expression of Bad, Bcl-xL, and C-caspase 3/caspase 3. Data are presented as the mean ± SEM, n = 3 per group, **p < 0.01 compared with the sham group, ^#^ *p* < 0.05, ^##^ *p* < 0.01 compared with the MCAO group.

We examined apoptosis in ischemic cortex using Bad, Bcl-xL, cleaved caspase 3 by western blotting. The results showed that expression of Bad, Bcl-xL, cleaved caspase 3 were significantly increased, compared to sham group (*p* < 0.01). Compare to MCAO group, QNDP decreased the expression of Bad and cleaved caspase 3, while increased the expression of Bcl-xL (*p* < 0.01). The results in QNDP+ NLRP3 inhibitor group as same as QNDP group, compared to MACO group (*p* < 0.01). The date are shown in **Figures 2A–C**. The above data are indicated that QNDP could inhibits cortical apoptosis.

### QNDP Inhibited NLRP3 Inflammasome Expression *In Vivo*

**Figure 3:**
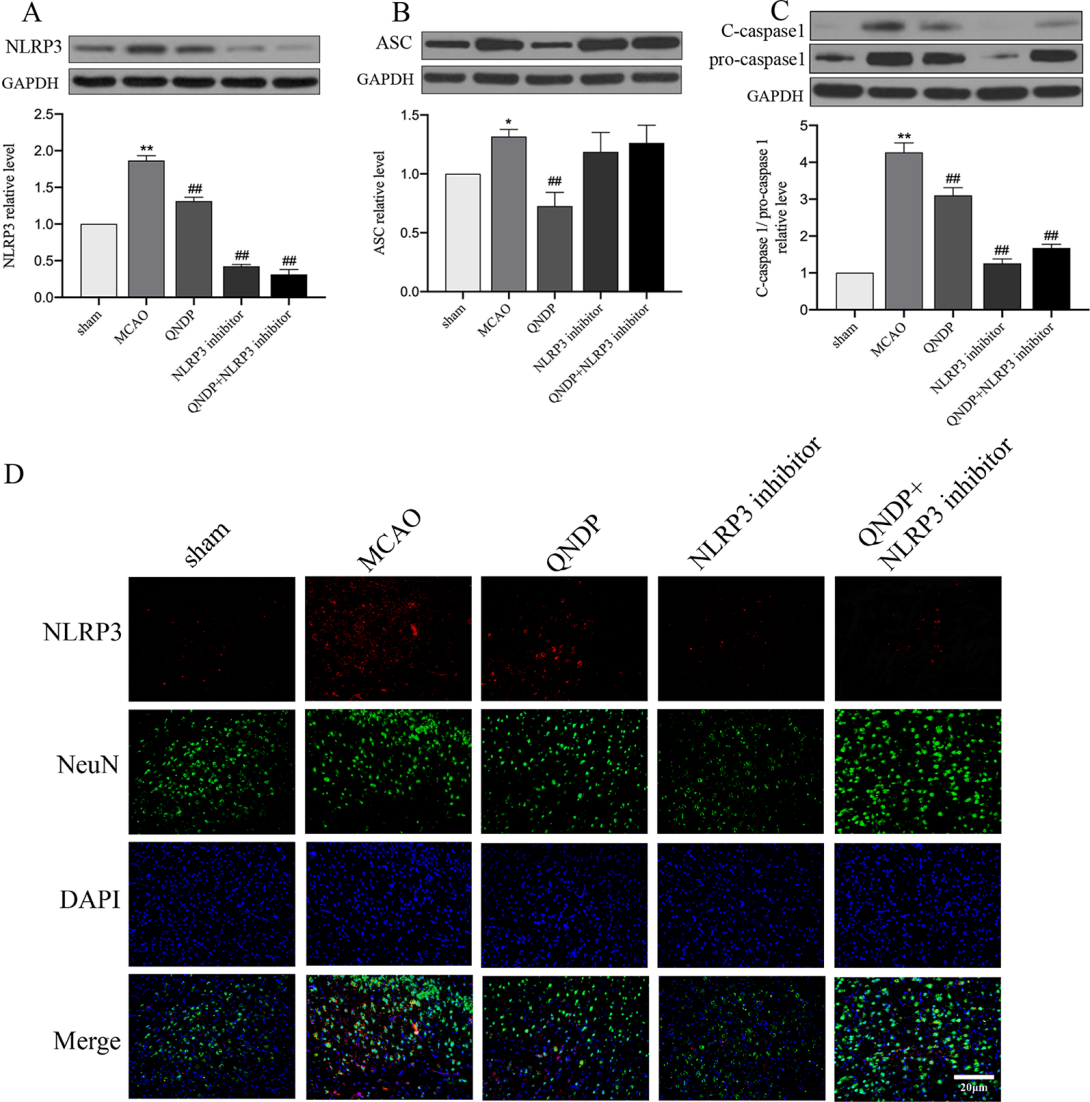
Effect of QNDP on the expression of NLRP3, ASC, and cleaved caspase 1/pro-caspase 1 in MCAO rats. **(A)** Western blotting analysis of NLRP3, **(B)** Western blotting analysis of ASC, **(C)** Western blotting analysis of C-caspase 1/pro-caspase 1, **(D)** Immunofluorescent staining for NLRP3 (red), NeuN (green), the nuclei were stained blue with DAPI. Scale bar indicates 20 μm. Data are presented as the mean ± SEM, n = 3 per group, *p < 0.05, **p < 0.01 compared with the sham group, ^##^ *p* < 0.01 compared with the MCAO group.

NLRP3 inflammasome has been demonstrated to be associated with neuroinflammation and cerebral ischemia, and plays an important role in cerebral ischemia and peaks 24 h after MCAO(Tang et al., 2010; Wang et al., 2019). To clarify whether QNDP inhibits NLRP3 inflammasome expression, we detected the expression of NLRP3 inflammasome at 24 h after MCAO following treatment with QNDP and sh-NLRP3 by immunofluorescence staining and western blotting. *In vivo*, as shown in **Figures 3A–C**, western blotting was detected the protein expression of NLRP3 inflammasome, ASC, cleaved caspase 1 in the ischemic cortex. The protein expression of NLRP3, ASC, and cleaved caspase 1 were significantly increased in the MCAO group, compared to the sham group (*p* < 0.01). QNDP treatment could significantly inhibit NLRP3, ASC, and cleaved caspase 1 expression (*p* < 0.01). However, compared to MCAO group, QNDP+NLRP3 inhibitor was significantly decreased the expression of NLRP3 and cleaved caspase 1 (*p* < 0.01), but it is interesting that NLRP3 inhibitor and QNDP+NLRP3 inhibitor has no significant with ASC. Furthermore, we examined NLRP3 expression in neurons in brain slices. Immunofluorescence staining showed that NLRP3 was mainly localized in the ischemic cortex. The images showed that the positive NLRP3 in QNDP and QNDP+NLRP3 inhibitor was less than those in MCAO group (**Figure 3D**). The **Figure 3D** was consistent with western blotting results. Double staining of NLRP3 and NeuN in brain slices demonstrated that NLRP3 partly co-localized with NeuN-immunoreactive neurons. In general, cerebral ischemia significantly elevated the expression of NLRP3 inflammasome, which was decreased by treatment with QNDP or QNDP+NLRP3 inhibitor. These results are indicated that QNDP may alleviate cerebral ischemia injury by inhibiting the NLRP3 inflammasome.

### QNDP Inhibited NLRP3 Inflammasome Expression *In Vitro*

**Figure 4:**
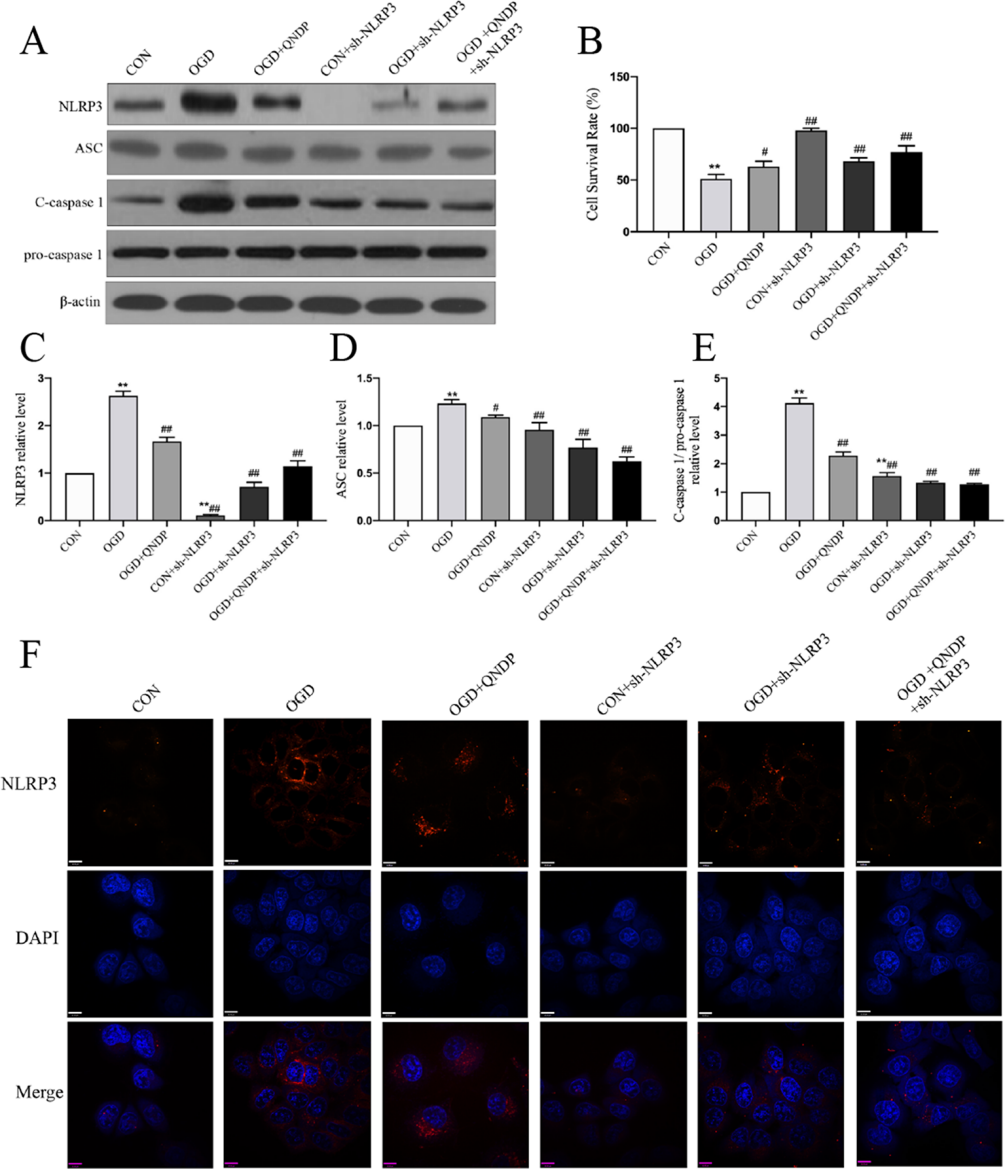
Effect of QNDP on the expression of NLRP3, ASC, and cleaved caspase 1 following OGD in SH-SY5Y cells. **(A)** Western blotting bands of NLRP3, ASC, C-caspase 1, pro-caspase 1, **(B)** Cell survival rate using CCK-8 assay. Western blotting analysis of NLRP3 **(C)**, ASC **(D)**, C-caspase 1/pro-caspase 1 **(E)**. **(F)** Immunocytochemical staining for NLRP3 (red), the nuclei were stained blue with DAPI. Scale bar indicates 10 μm. Data are presented as the mean ± SEM, n = 3-5 batches of cells, **p < 0.01 compared with the sham group, ^#^ *p* < 0.05, ^##^ *p* < 0.01 compared with the MCAO group.

To determine whether the effects of QNDP on NLRP3 inflammasome activated in SH-SY5Y cells under OGD, we analyzed expression of NLRP3 inflammasome *in vitro* by western blotting and immunofluorescence staining. As shown in **Figure 4**, the data showed an increase in protein expression of NLRP3, ASC, and cleaved caspase1 during OGD. Compared to OGD group, QNDP and QNDP+NLRP3 inhibitor were obviously decreased the protein expression of NLRP3, ASC, and cleaved caspase 1 (*p* < 0.05 or 0.01, **Figures 4 A–E**). Next, we detected cell viability using CCK-8 assay. The data showed that OGD leads to a significant reduction in cell viability (*p* < 0.01, **Figure 4B**), while QNDP and QNDP+sh-NLRP3 were significantly increased *via* OGD group *(p* < 0.01). To further confirm the expression and activation of the NLRP3, we observed NLRP3 levels in the SH-SY5Y cells by immunofluorescence staining. Images showed NLRP3 appeared in the cytoplasm, and its fluorescence intensity was significantly enhanced by OGD (**Figure 4F**). QNDP or QNDP+sh-NLRP3 was significantly reduced the fluorescence intensity. These results are consistent with those derived from western blotting. The above data are shown that QNDP may inhibit the NLRP3 inflammasome in SH-SY5Y cells.

### QNDP Decreased the Inflammatory Cytokines Level by Inhibiting IL-1β, and IL-18 After Cerebral Ischemia *In Vivo*

**Figure 5:**
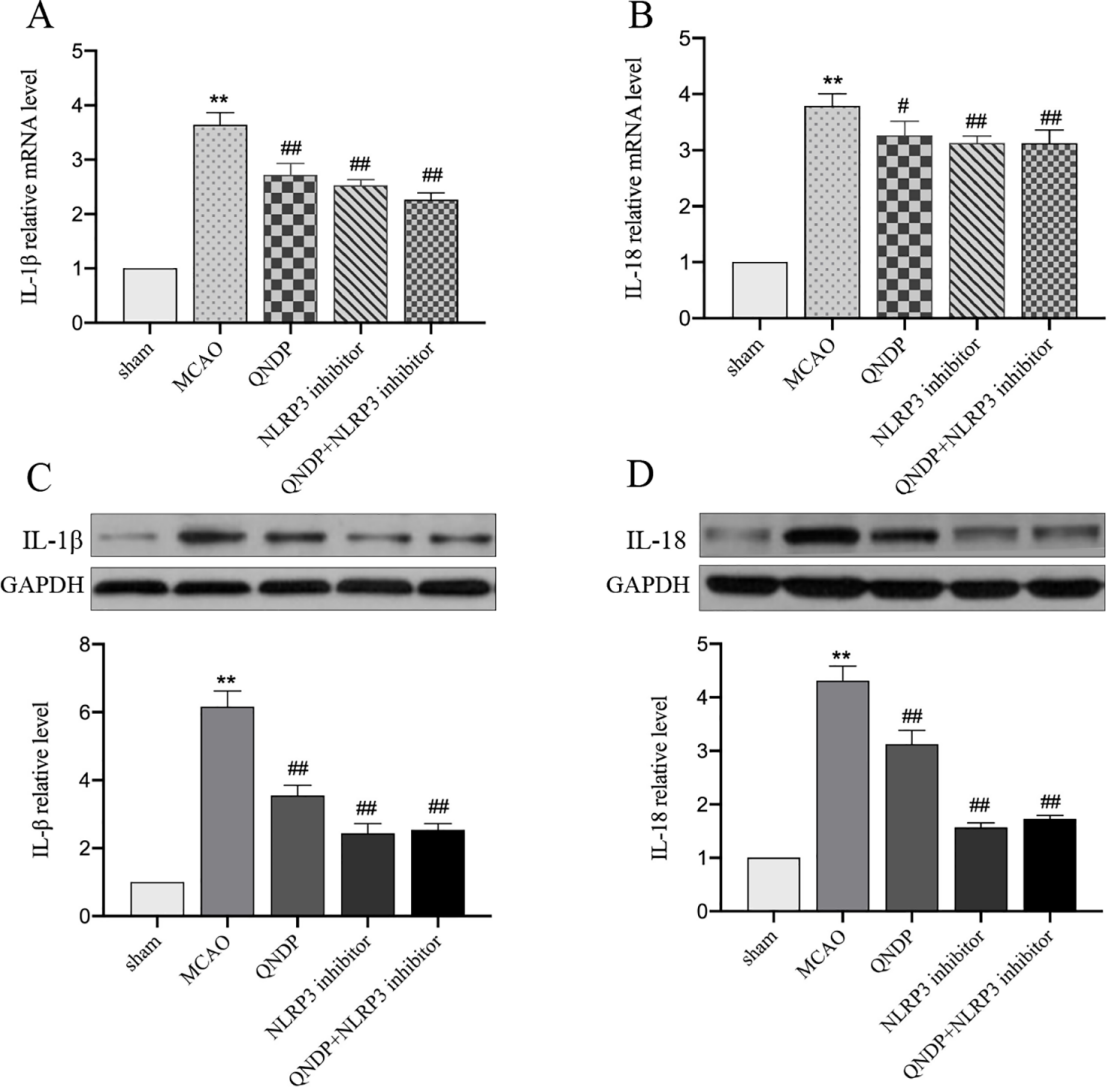
Effect of QNDP on the expression of IL-1β and IL-18 in MCAO rats. Real- time PCR analysis of IL-1β **(A)**, IL-18 **(B)**. n = 8 per group. Western blotting analysis of IL-1β **(C)**, IL-18 **(D)**, n = 3 per group. Data are presented as the mean ± SEM, **p < 0.01 compared with the sham group, ^#^ *p* < 0.05, ^##^ *p* < 0.01 compared with the MCAO group.

It is known that NLRP3 inflammasome activation promotes the secretion of IL-1β, IL-18 (He et al., 2017). We next examined whether QNDP affects effectors downstream of the NLRP3 inflammasome. To determine the effect of QNDP on pro-inflammatory cytokines after cerebral ischemia in rats, we detected the mRNA levels of IL-1β, IL-18 in the ischemic cortex by real time PCR (**Figures 5A, B**). Compared to the sham group, the mRNA levels of IL-1β and IL-18 were significantly increased in MCAO group (*p* < 0.01). In the QNDP group, the mRNA levels of IL-1β and IL-18 were significantly decreased (*p* < 0.05 or 0.01). When QNDP add NLRP3 inhibitor group, the results as same as QNDP alone. There was no statistical difference between the QNDP group and the QNDP+NLRP3 inhibitor group (*p* > 0.05). Similar to the results of real time PCR results, those of western blotting data were showed that the expression of IL-1β and IL-18, were obviously higher in the MCAO group than in the sham group (*p* < 0.01). QNDP and QNDP+NLRP3 inhibitor proudly decreased the expression of IL-1β and IL-18 (*p* < 0.01, **Figures 5C, D**). These findings are suggested that QNDP may alleviate cerebral ischemia injury by inhibiting the NLRP3 inflammasome-mediated release of cytokines, such as IL-1β, and IL-18.

### QNDP Inhibited NF-κB Pathway After Cerebral Ischemia *In Vivo*

**Figure 6:**
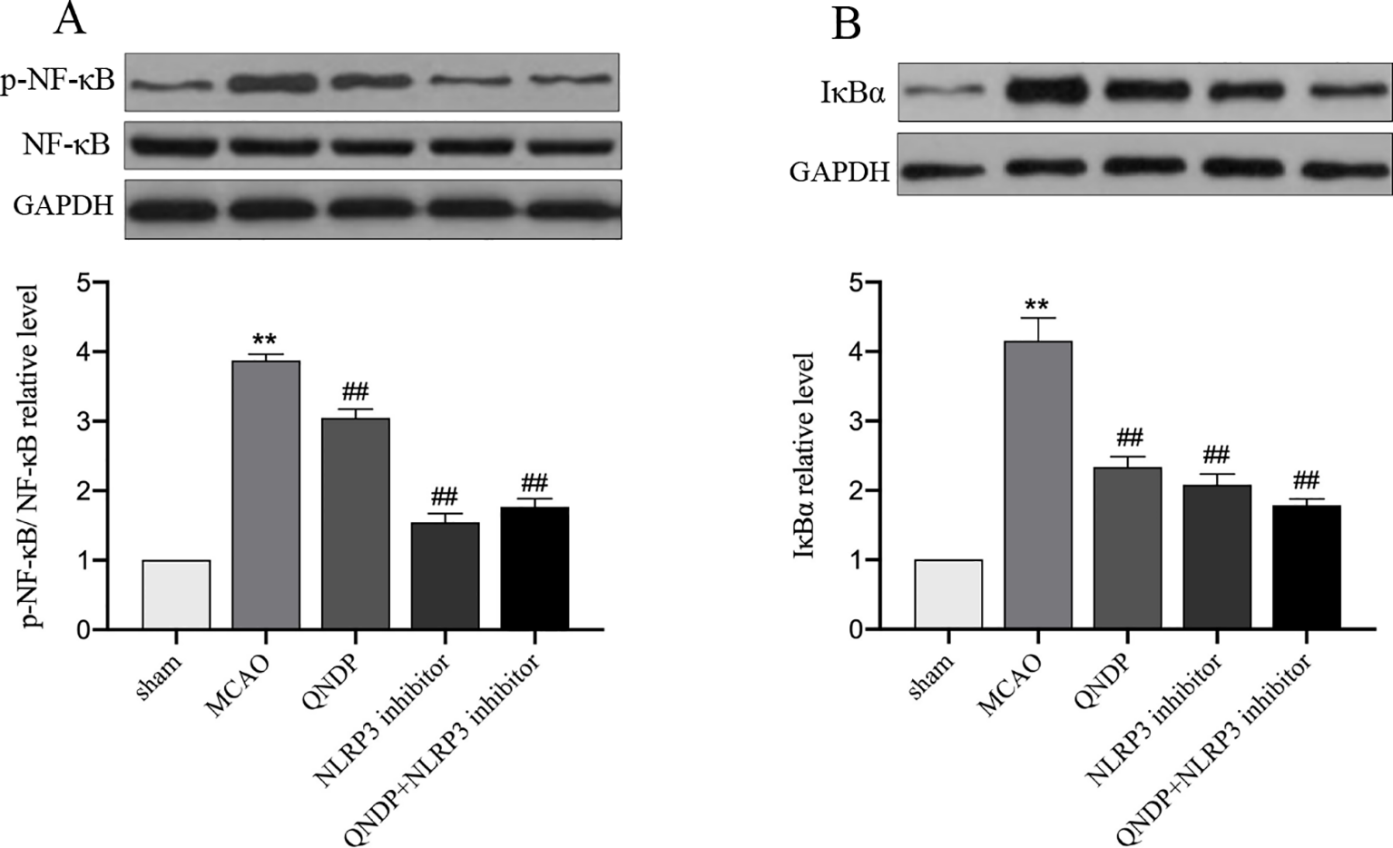
Effect of QNDP on the protein expression of p-NF-κB, NF-κB and IκBα in MCAO rats. Representative immunoblots and quantification illustrating the effect of QNDP on protein expression of p-NF-κB/NF-κB and IκBα. Data are presented as the mean ± SEM, n=3 per group, **p < 0.01 compared with the sham group, ^##^ *p* < 0.01 compared with the MCAO group.

It is widely known that mRNA transcription of inflammatory cytokines depends on NF-κB activation. The priming step of NLRP3 inflammasome activation triggered by activating NF-κB signaling which induces NLRP3 transcription (He et al., 2019b). To further test the hypothesis that the effects of QNDP on the regulation of NLRP3 inflammasome activation are mediated by NF-κB. Since IL-1β, IL-18, and NLRP3 inflammasome were up-regulated in the cortex after MCAO and obviously decreased by QNDP, we detected the expression of phosphorylation protein of NF-κB p65 (p-NF-κB) and IκBα in ischemic cortex. As shown in **Figure 6**, compared to the sham group, the p-NF-κB, and IκBα expression were significantly increased in the MACO group (*p* < 0.01, **Figures 6A, B**), which were markedly decreased by QNDP or QNDP+NLRP3 inhibitor, compared to the MCAO group (*p* < 0.01). These results are suggested that QNDP inhibiting NLRP3 inflammasome activation and cleaved-caspase 1, IL-1β, and IL-18 levels may be related with suppressing NF-κB pathway.

## Discussion

The present study generated three findings: (i) the important contribution of inflammatory response and apoptosis in cerebral ischemia injury, (ii) the anti-inflammatory and anti-apoptotic effect of QNDP in cerebral ischemia injury with evidence from both *in vivo* and *in vitro* models, and (iii) the crucial role of NLRP3 signaling pathway in mediating inflammatory response and apoptosis as an underlying mechanism for the effect of QNDP. These results suggest that QNDP is a potential candidate for the future treatment of ischemic stroke.

Stroke is a major cause of death and disability worldwide, and inflammation and apoptosis are key factors that cause and promote the cerebral ischemia injury (Lakhan et al., 2009). Importantly, apoptosis contributes to a significant proportion of neuron death in cerebral ischemia (Radak et al., 2017). The previous studies have illustrated that neuron apoptosis was initiated by activation of caspase molecules(Nijboer et al., 2008), and the inhibition of caspase 3 could prevent neuron apoptosis to protect against cerebral ischemia injury (Wang et al., 2014b). Besides, Bcl-2 family proteins are also critical in apoptosis, some members of which inhibit apoptosis (such as Bcl-2 and Bcl-xL), whereas others promote cell death (such as Bax and Bad) (Youle and Strasser, 2008; Jonas et al., 2014). A number of studies have suggested that Bcl-2 family proteins involved in the occurrence and development of stroke and played a role as a regulator of the apoptosis pathway in cerebral ischemia (Martinou et al., 1994; Hata et al., 1999; Wiessner et al., 1999; Qi et al., 2015). In this study, QNDP reduced infarct volume in MCAO rats, the same as the results of our previous study (Zeng et al., 2019). In addition, QNDP increased levels of Bcl-xL, and decreased levels of Bad and caspase 3, which meant QNDP alleviated apoptosis in cerebral ischemia injury. Our results suggested that QNDP can effectively protect against ischemic stroke by inhibiting apoptosis.

It is known that the inflammatory process is inherent across the whole time course of stroke(Iadecola and Anrather, 2011; Barrington et al., 2017), and understanding inflammatory mechanism can be helpful and meaningful for finding potential treatment opinions(Jayaraj et al., 2019). Following the acute stroke, sterile inflammation brings about the production of pro-inflammatory cytokines, such as IL-1β and IL-18, which exacerbates brain damage through activation of resident cells (i.e., microglia) and recruitment of inflammatory cells (Barrington et al., 2017; Hao et al., 2019; Jayaraj et al., 2019). Several studies have found that inhibition of IL-1β could cause significantly decreases in infarct volume (up to 60% to 70%) in MCAO mice and rats (Boutin et al., 2001; Touzani et al., 2002; Lambertsen et al., 2012), and increased level of IL-18 may contribute to the development and severity of ischemic stroke (Hao et al., 2019).This study referred that QNDP therapy obviously decreased the protein and mRNA expression of IL-1β and IL-18, and further demonstrated the notable intervention of QNDP on inflammatory response in cerebral ischemia based on our precious study(Zeng et al., 2019).

In recent years, there has been a growing interest in the role of inflammasome in cerebral ischemia. Inflammasome as a newly identified pattern-recognition receptors, was firstly described in detail by Martinon in 2002 (Martinon et al., 2002; Shao et al., 2015). It has been identified numerous inflammasomes, such as including NLRP3, NLRP1, NLRC4, AIM2, and so on(Yang et al., 2019). Among them, the NLRP3 inflammasome is one of most widely in the research. The NLRP3 inflammasome comprises the sensor molecule NLRP3, the adaptor protein ASC, and pro-caspase 1. Once activation, the NLRP3 protein interacts with ASC through the PYD domain, then the CARD domain of ASC recruits and activates pro-caspase 1, to form NLRP3–ASC–pro-caspase-1 complex (Guo et al., 2015). NLRP3 inflammasome acts a principal role in the pathogenesis of various inflammatory diseases. The studies reported that the activation of the NLRP3 inflammasome leads to the development and release of inflammatory cytokines IL-1β and IL-18, suggesting the role for the NLRP3 inflammasome in the initiation and development of cerebral ischemia (Rathinam et al., 2012; Gao et al., 2017). Accumulating amounts of evidence have indicated that NLRP3 inflammasome activation was closely related with post-ischemic inflammation after stroke, which was critical in neuronal cell death in ischemic stroke (Zhang et al., 2017; Alishahi et al., 2019).

The studies found that the levels of NLRP3 inflammasome proteins, IL-1β and IL-18 were elevated in stroke patients, and also increased in ipsilateral brain tissues of MCAO C57BL/6J mice and primary cortical neurons exposed to OGD (Fann et al., 2013; Wang et al., 2014a). The study found the highest level of NLRP3 inflammasome occurred at 24 h after ischemia–reperfusion injury (Hou et al., 2018). Gong et al. found that NLRP3 inflammasomes were first activated in microglia soon after cerebral ischemia injury onset and then were expressed in microvascular endothelial cells and neurons later, but they were mainly in neurons(Gong et al., 2018). Based on the above results, it can be deduced that NLRP3 inflammasome were rapidly activated and simultaneously activated inflammation, leading to neurons damaged after ischemic injury. When NLRP3 inflammasome was upregulated, following also IL-1β and IL-18 increased, and neurons are partly damaged from the ischemic insult. Consistent with these reports, our data demonstrate NLRP3, ASC and cleaved caspase 1 were substantially upregulated in the ischemic cortex in MCAO rats and SH-SY5Y cells exposed to OGD. In addition, IL-1β and IL-18 protein and mRNA levels in the ischemic cortex were also markedly elevated *in vivo*. Conversely, when NLRP3 inflammasome was blocked, neurons progress toward better. It was clarified that that NLRP3 inflammasome is harmful in ischemic stroke again. Hou et al. had reported that NLRP3 siRNA was reduced the infarct volume and improve neurological deficit, may be related with decrease the expression of the NLRP3 inflammasome and downstream IL-1β, and IL-18 of inflammation factors (Hou et al., 2018). Another research from Yu et al. reported that the inhibition of NLRP3 inflammasome activation, which was reduced infarct volumes, decreased edema formation, has a neuroprotective effect in cerebral ischemia injury(Yu et al., 2017). These data suggested that NLRP3 inflammasome might be an inflammation contributor to cerebral ischemia injury. Those studies were in accordance with our results. We using NLRP3 shRNA or QNDP found that QNDP or QNDP and sh-NLRP3 obviously inhibited the levels of NLRP3, ASC and cleaved-caspase 1, then to regulate the inflammatory cytokines and inhibit apoptosis in cerebral ischemia *in vivo* and *in vitro*. In addition, we found that not all NeuN-immunoreactive neurons in the brain slices expressed NLRP3 after cerebral ischemia, which is worth exploring in depth. In our present study, QNDP could reduce the expression of NLRP3, cleaved caspase-1, IL-1β, and IL-18, alleviate brain edema, and improve the neurological function after cerebral ischemia. These results indicated that QNDP protects the neurons and alleviates brain injury against inflammation through a mechanism mediated by NLRP3 inflammasome partly.

NF-κB signal pathway is considered to be a vital regulator of inflammation response and is critical to the regulation of apoptosis, plays the important role in ischemic stroke (Harari and Liao, 2010; Li et al., 2019; Liu et al., 2019). NF-κB further promotes the production of NLRP3 and IL-1β and enhancing inflammation (Minutoli et al., 2016; Chen et al., 2019). Among various studies, NF-κB could regulate NLRP3 inflammasome activity (Pan et al., 2018). Fann et al. provided the results that both the NF-κB and MAPK signaling pathways play a pivotal role in regulating the expression and activation of NLRP3 inflammasomes in primary cortical neurons and brain tissue under ischemic conditions (Fann et al., 2018). In our previous study, we had illustrated activation MAPK signaling pathways was partly responsible for inducing cerebral ischemia. QNDP could inhibit MAPK pathway to alleviate cerebral ischemia injury. We focused on NLRP3 inflammasome in the current study to explore the relationship between NF-κB and the NLRP3 inflammasome in cerebral ischemia injury. We observed that the activation of NF-κB was increased, expression of the NLRP3 inflammasome also elevated, and the levels of downstream inflammatory factors as IL-1β, and IL-18. QNDP or QNDP+sh-NLRP3 had an opposite tendency.

## Conclusion

QNDP displayed an inhibitory activity on the activation of NLRP3 inflammasome in MCAO rats and SH-SY5Y cells exposed to OGD. QDNP also inhibited the levels of mRNA and protein expression of IL-1β, and IL-18 in MCAO rats. Above all, the research suggests that QNDP shows an inhibitory activity on blocking the activation of NLRP3 inflammasome *in vivo* and *in vitro*, may be related with suppressing NF-κB pathway. QDNP significantly decreases cerebral ischemia injury and improve neurological function, protects the neurons against inflammation through a mechanism mediated by NLRP3 signaling. Furthermore, we want to do some more study to clarify which active components of QNDP plays the key role with NLRP3 inflammasome.

## Data Availability Statement

The datasets generated for this study can be available on request to the corresponding author.

## Ethics Statement

The animal study was reviewed and approved by the Animal Care and Use Committee of Dongfang Hospital, Beijing University of Chinese Medicine.

## Author Contributions

CF performed the experiment and wrote the manuscript. XZ and ZZ assisted in the *in vivo* experiments and data analysis. YT and ZX assisted in the *in vitro* experiments. FW and HZ supervised the experiments. XJ and BC assisted in data analysis. XL conceived the study, designed the experiments and wrote the manuscript. All the authors reviewed and approved the manuscript.

## Funding

This work was supported by the Natural Science Foundation of China (No. 81973788), the Beijing National Science Foundation (No.7182099), the Natural Science Foundation of China (No.81202683) and the International Science and Technology Cooperation Program of China (No. 2015DFA31130).

## Conflict of Interest

The authors declare that the research was conducted in the absence of any commercial or financial relationships that could be construed as a potential conflict of interest.
