# Supplementary material for: Neuroprotective Effects of Qingnao Dripping Pills Against Cerebral Ischemia via Inhibiting NLRP3 Inflammasome Signaling Pathway: In Vivo and In Vitro
Source: Front Pharmacol. 2020 Feb 20;11:65. doi: 10.3389/fphar.2020.00065 (PMC7045811; doi:10.3389/fphar.2020.00065)
Supplement: Supplementary file 1 [file DataSheet_1.docx]

**Supplementary Material**

**Neuroprotective Effects of** **Qingnao Dripping Pills** **against** **cerebral ischemia via inhibiting****NLRP3 inflammasome signaling pathway: *in Vivo* and *in Vitro***

Legend to figures and Tables


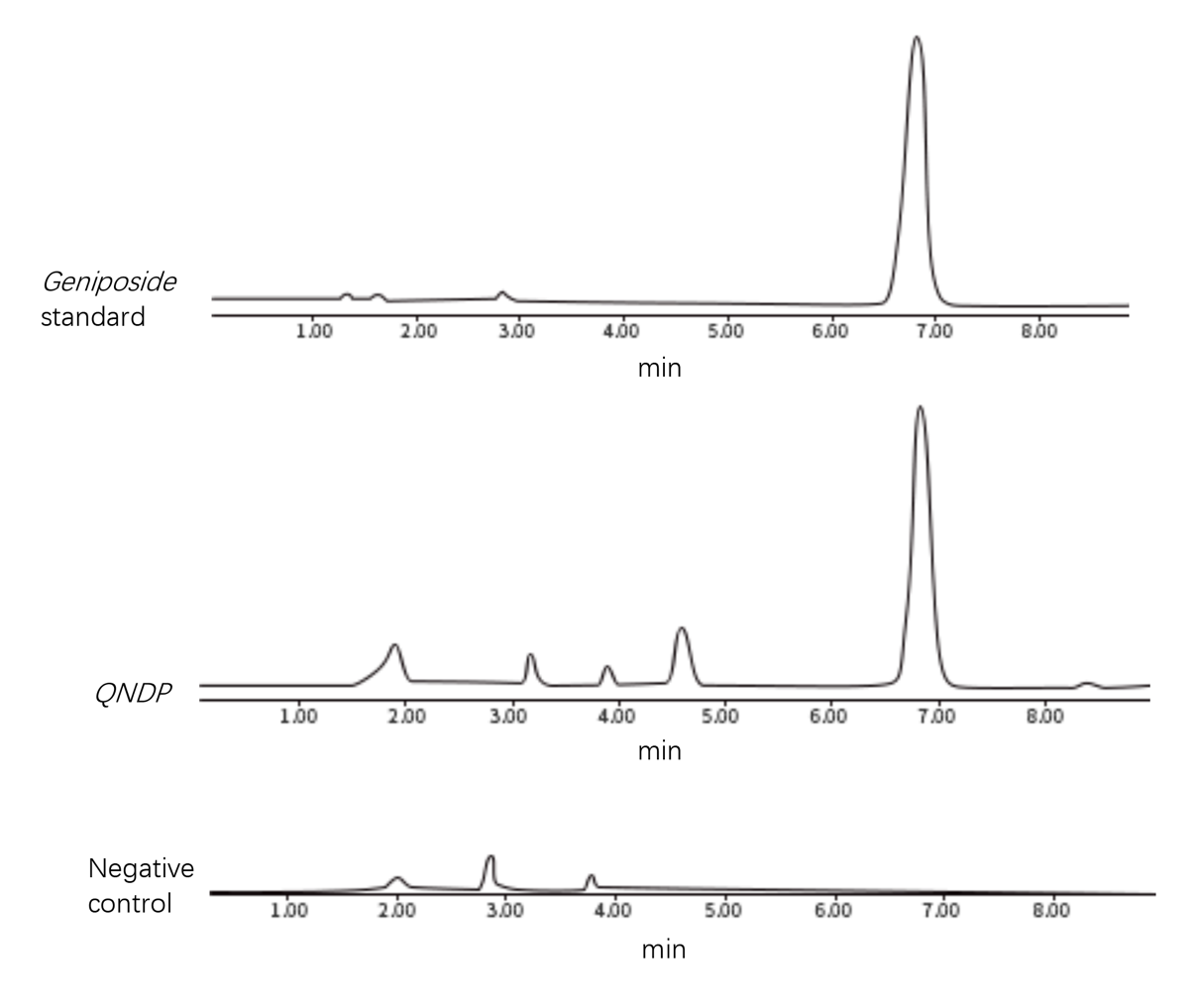


**Supplementary FIGURE 1**  HPLC chromatogram of Geniposide standards and QNDP.


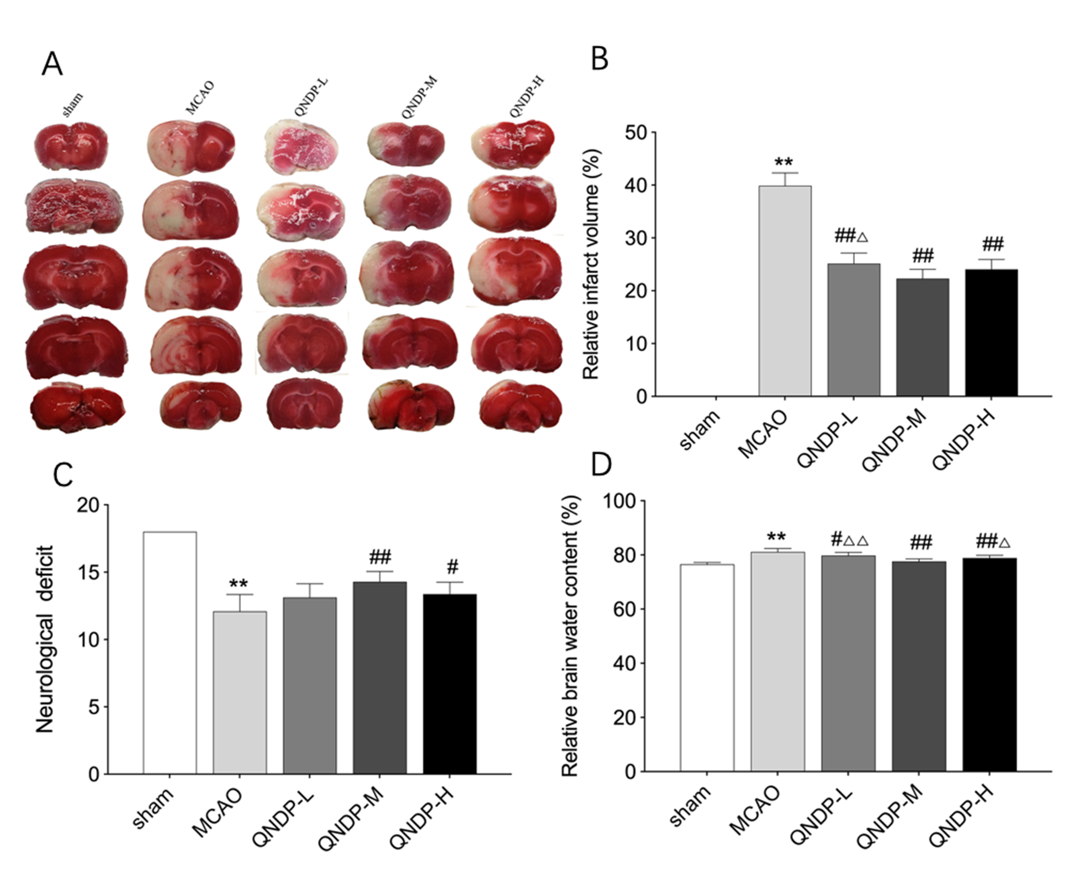


**Supplementary FIGURE 2**  neuroprotective effect on fifferent dose of QNDP in MCAO rats. QNDP-L: 0.08 g/kg, QNDP-M:0.15 g/kg, QNDP-H:0.30 g/kg. (A) Images of cerebral infarction (white, infarct tissue; red, non-infarct tissue) were TTC-stained brain sections. (B) The infarct volume was quantified with Image J analysis and expressed as a percentage of the damaged ipsilateral hemisphere. (C) Neurological deficit by Garcia JH scores. (D) Brain water content. The results showed MCAO were significantly increased in the infarct volume and brain water content weter, decreased in neurological deficit, compared to control group (**P < 0.01). Compared with the MCAO group, QNDP-M and QNDP-H were obviously increased in the infarct volume and brain water content and decreased in the neurological deficit (^##^P < 0.01). Compared with QNDP-M group, QNDP-H had a higher brain water content (^△^P<0.01). Data are presented as means ± SEM, n=8 per group, **p < 0.01 compared with the sham group, ##p < 0.01 compared with the MCAO group.


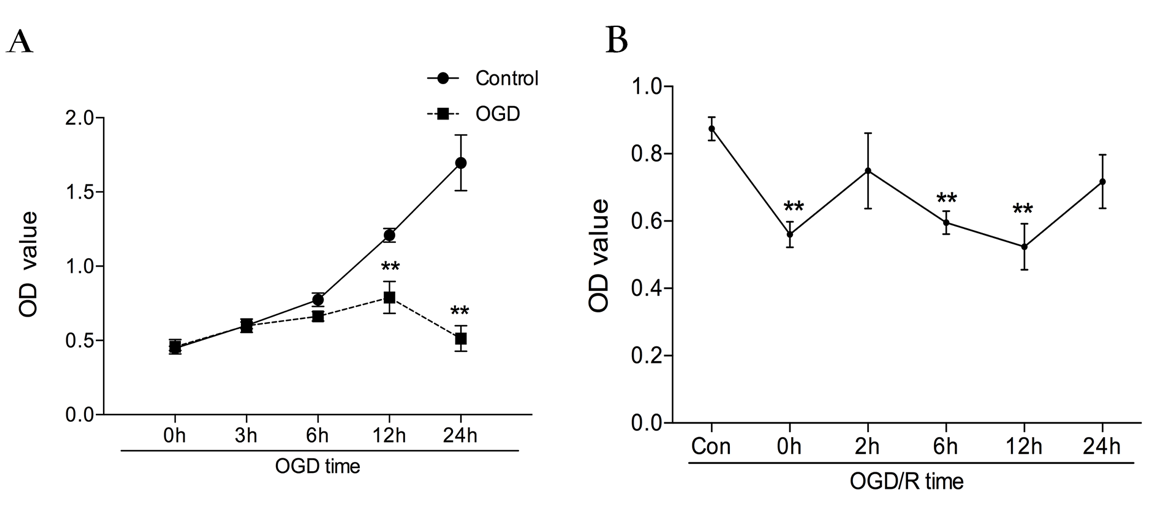


**Supplementary FIGURE 3** Time-dependent effect of OGD and appropriate time span of reoxygenation in the growth of SH-SY5Y cells. A, MTT assay showed that the growth of the SH-SY5Y cells was significantly decreased between 12 h group and 24 h group after OGD, compared to OGD 0 h group (**P < 0.01). B, The results showed that the growth of SH-SY5Y cells would further decreased after 6 h group and 12 h group of reoxygenation, compared to 0 h group of reoxygenation (**P < 0.01). Data are expressed as the means±S.E.M (n =12/group).


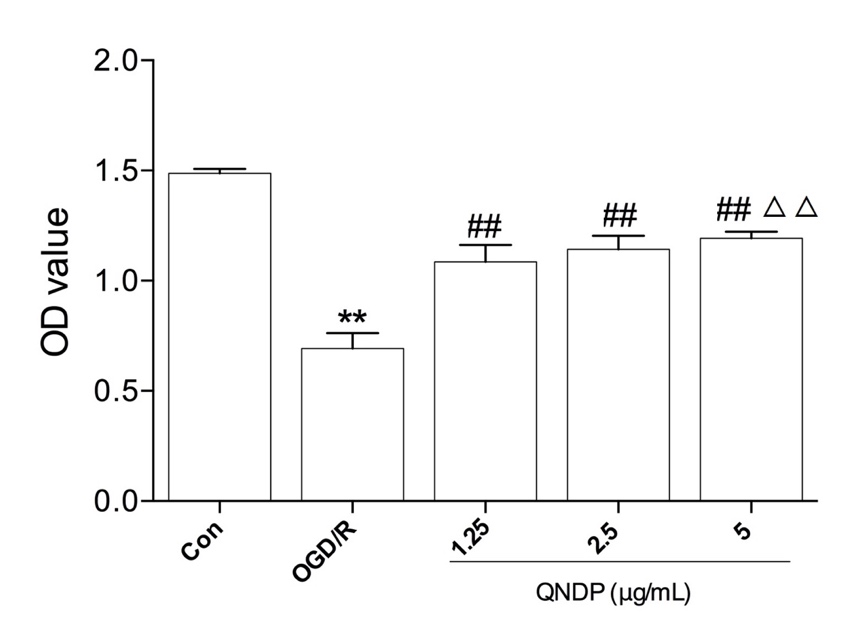


**Supplementary FIGURE 4** Dose-dependent effect of QNDP on the growth of SH-SY5Y cells in an OGD/R model. MTT assay showed the growth of SH-SY5Y cells in OGD/R groups was significantly decreased compared to control group (^##^P < 0.01). Compared with the OGD/R group, the 1.25 μg/mL QNDP group, 2.5 μg/mL QNDP group, and 5 μg/mL QNDP group had an increase in cell growth (**P < 0.01). Compared with the 1.25 μg/mL QNDP group, the 5 μg/mL QNDP group had a higher cell growth (^△△^P<0.01). Data are expressed as the means ± S.E.M (n=12/group).


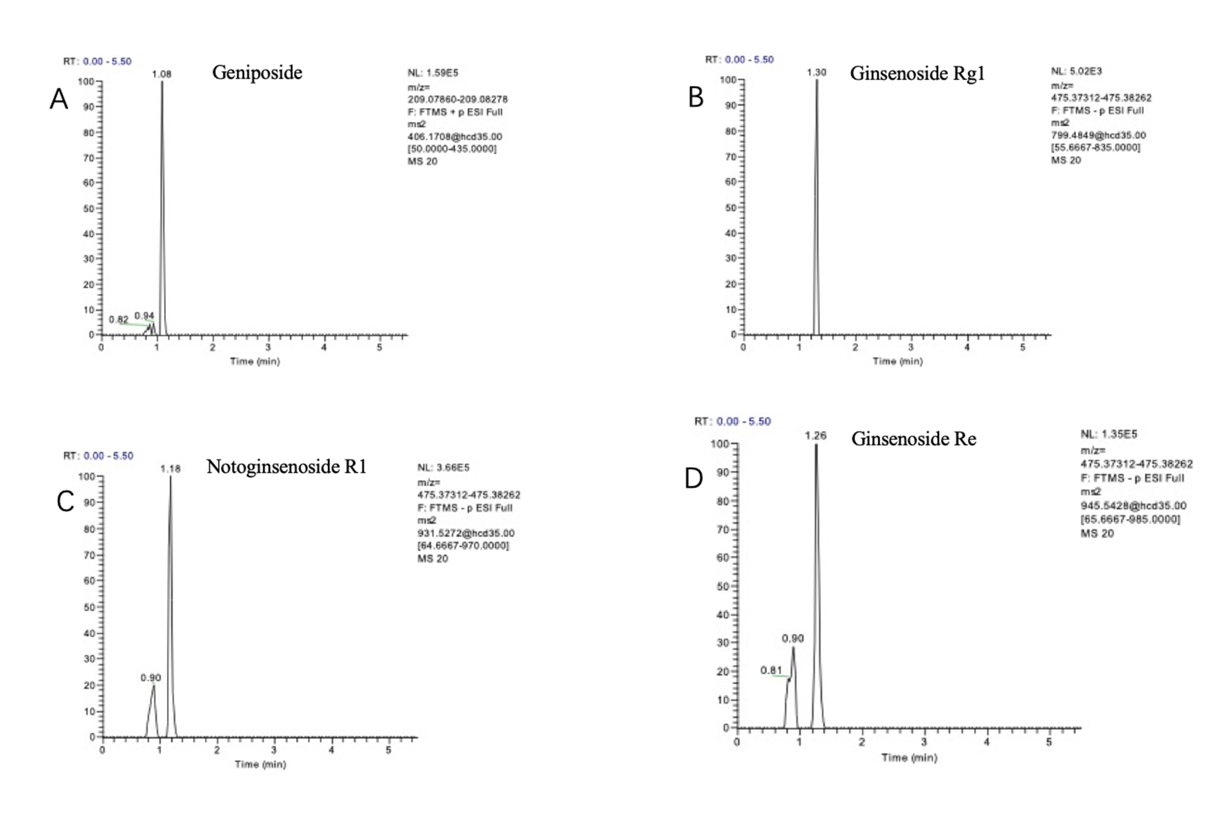


**Supplementary FIGURE 5** Chromatograms of the four main components of QNDP by LC/MS. A. Geniposide; B. Ginsenoside Rg1; C. Notoginsenoside R1; D. Ginsenoside Re


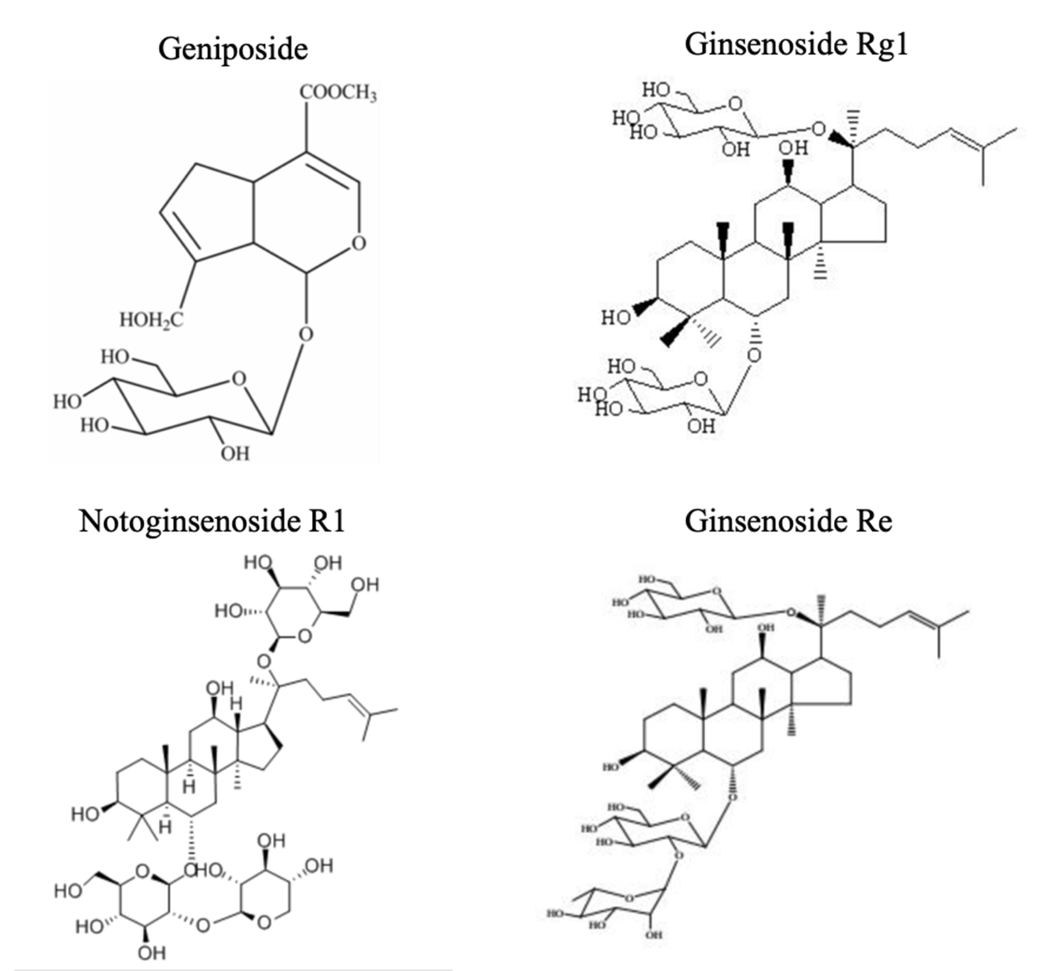


**Supplementary FIGURE 6** The chemical structures of the main components of QNDP

**Supplementary TABLE 1** Detailed information of the crude drugs of QNDP.

| **Voucher number** | **Drug name** | **Authentication** | **Voucher specimen** |
| --- | --- | --- | --- |
| 18-BUCM-Q3-1 | Chaozhizi | Fruit of *Gardenia jasminoides Ellis* | 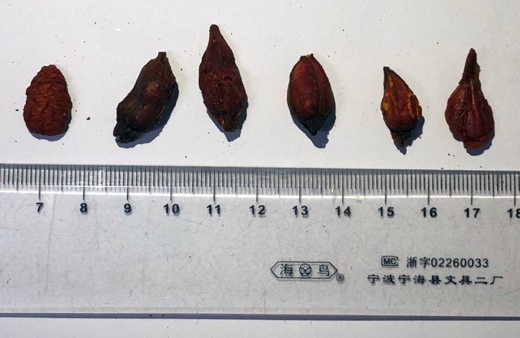 |
| 18-BUCM-Q1-2 | Sanqi | Root of *Panax notoginseng (Burk.) F.H.Chen* | 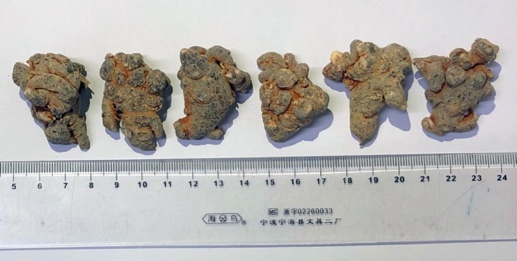 |
| 18-BUCM-Q4-3 | Bingpian | Borneol | 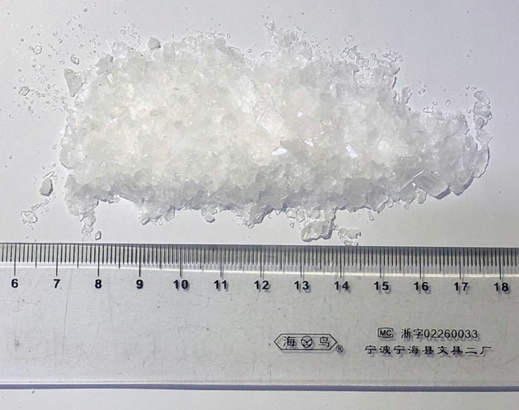 |

**Supplementary TABLE 2** Concentrations of the four main

components of QNDP (n=3, mg/g)

|  | Concentrations |
| --- | --- |
| Geniposide | 145.6±18.86 |
| Ginsenoside Rg1 | 77.1±2.4 |
| Notoginsenoside R1 | 5.8±0.2 |
| Ginsenoside Re | 4.9±0.3 |

Concentrations of the main components of QNDP, determined by LC-MS analysis.
